# Scale Effect of Land Cover Classification from Multi-Resolution Satellite Remote Sensing Data

**DOI:** 10.3390/s23136136

**Published:** 2023-07-04

**Authors:** Runxiang Li, Xiaohong Gao, Feifei Shi, Hao Zhang

**Affiliations:** 1 School of Geographical Sciences, Qinghai Normal University, Xining 810008, China; lrx7471870@163.com (R.L.); shifeifei1203@126.com (F.S.); 15959785022@163.com (H.Z.); 2 Qinghai Province Key Laboratory of Physical Geography and Environmental Process, Xining 810008, China; 3 Ministry of Education Key Laboratory of Tibetan Plateau Land Surface Processes and Ecological Conservation, Xining 810008, China; 4 Academy of Plateau Science and Sustainability, Xining 810008, China

**Keywords:** land cover, scale effect, uncertainty, spatial heterogeneity

## Abstract

Land cover data are important basic data for earth system science and other fields. Multi-source remote sensing images have become the main data source for land cover classification. There are still many uncertainties in the scale effect of image spatial resolution on land cover classification. Since it is difficult to obtain multiple spatial resolution remote sensing images of the same area at the same time, the main current method to study the scale effect of land cover classification is to use the same image resampled to different resolutions, however errors in the resampling process lead to uncertainty in the accuracy of land cover classification. To study the land cover classification scale effect of different spatial resolutions of multi-source remote sensing data, we selected 1 m and 4 m of GF-2, 6 m of SPOT-6, 10 m of Sentinel-2, and 30 m of Landsat-8 multi-sensor data, and explored the scale effect of image spatial resolution on land cover classification from two aspects of mixed image element decomposition and spatial heterogeneity. For the study area, we compared the classification obtained from GF-2, SPOT-6, Sentinel-2, and Landsat-8 images at different spatial resolutions based on GBDT and RF. The results show that (1) GF-2 and SPOT-6 had the best classification results, and the optimal scale based on this classification accuracy was 4–6 m; (2) the optimal scale based on linear decomposition depended on the study area; (3) the optimal scale of land cover was related to spatial heterogeneity, i.e., the more fragmented and complex was the space, the smaller the scale needed; and (4) the resampled images were not sensitive to scale and increased the uncertainty of the classification. These findings have implications for land cover classification and optimal scale selection, scale effects, and landscape ecology uncertainty studies.

## 1. Introduction

Remote sensing provides data on a large scale and around the clock for use in various industries. Land cover classification data obtained by remote sensing are essential data for studying surface processes and for climate model simulations [1,2,3,4]. In recent decades, with the development of space science and multi-platform remote sensing, multi-sensor and multi-angle technologies, the spatial resolution, spectral resolution, and temporal resolution of remote sensing images have been improving. Spatial resolution is one of the basic characteristics of remote sensing images, and the scale effect in remote sensing is a key research problem. Woodcock [5] considered that spatial resolution should be similar to the scale of observation. Multi-source satellite remote sensing images have become the basic data source for regional, national, and global mapping. Studies have shown that land cover mapping is influenced by the spatial resolution of remote sensing images, which has an obvious spatial scale effect [6,7,8]. The scale effect of the information acquired by remote sensing is the key to obtaining optimal scale land cover mapping based on the optimal resolution for a particular study area. Han et al. [9] used the method of information entropy to solve the average entropy of category differentiability of image data at each scale and calculate the optimal scale. They showed that the optimal scale has a relationship with the spatial distribution characteristics of the features. Treitz [10] used the variance function to calculate the optimal spatial resolution based on the theory of spatial autocorrelation analysis of spatial statistics and concluded that the optimal scale was related to the ground scene and sensor parameters. Dongping Ming et al. [11] proposed an improved local variance method based on the variable window and variable resolution to determine the optimal resolution using local variance as a measure. Their results are not applicable to a large range of complex environments. Feng et al. [12] used a Triangular Prism Method and Double Blanket Method to determine the resolution of images with three fractal dimensions, and the results had uncertainties. Ming et al. [13] studied the optimal spatial resolution of different features in remote sensing images by using the improved method of average local variance and concluded that variance increases with increasingly complex feature information. The above studies are based on the optimal spatial resolution calculated by geostatistical and classical statistical methods, and there is a large uncertainty in the research conclusions. Research on the scale effect of remote sensing images still lacks clear conclusions.

Generally speaking, features have inherent scales, and the expression at the inherent scale is the most realistic representation of the features [1,14]. However, it is difficult to obtain images with different spatial resolutions from multiple satellites covering the same area due to weather conditions, cloud cover, sensor performance, satellite transit times, and other factors, so most current studies of scale effects often use the same image resampled to obtain data with different spatial resolutions [6,15,16,17,18,19,20,21,22]. However, due to the existence of spatial heterogeneity, resampling can cause distortion of features and loss of spectral information, and the resampled images are different from the real satellite images at a specific spatial scale. Therefore, the results of these studies are still somewhat questionable [23,24].

Markham and Townshend [25] argued that remote sensing classification accuracy is mainly influenced by two factors. The first factor is the image elements at the edge between categories in the classification results, i.e., the hybrid image elements. When the spatial resolution of the image increases, the number of hybrid image elements at the edge between different ground feature categories decreases, and the classification accuracy increases. The second factor is spatial heterogeneity. When the spatial resolution increases, the variability of spectral features within the same feature category increases, which causes the inter-category separability to decrease, thus leading to a decrease in classification accuracy. On the surface, the accuracy effects of spatial resolution variation in relation to mixed image elements and spatial heterogeneity are contradictory. However, the variation in classification accuracy ultimately still depends on the relative relationship between the spatial resolution of the image and the size of the target within the scene. For larger homogeneous targets, the reduction in spatial resolution only increases the number of hybrid pixels at the edges but does not cause a change of spectral variability between pixels within the target, so the classification accuracy is reduced. In contrast, for targets with large spectral-spatial heterogeneity, the reduction in spatial resolution increases the number of hybrid pixels at the edges, but the smoothing effect of the reduced spatial resolution may improve the accuracy of the final classification results, which may reduce the intra-class spectral variation and increase the distinguishability between classes. Woodcock and Strahler [5] argued that the net effect of these two conflicting factors is a function of the environment of the image scene. Therefore, it is necessary to analyze the effect of spatial resolution variation on land cover classification accuracy based on multi-source remote sensing data in terms of both mixed image element decomposition and spatial heterogeneity and to study the optimal scale effect of land cover classification at different spatial resolutions from multi-source remote sensing data.

In this study, we selected 1 m and 4 m data from GF-2 satellite, 6 m data from SPOT-6, 10 m data from Sentinel-2, and 30 m data from Landsat-8 OLI to quantitatively investigate the relationship between land cover classification results and different spatial resolution from multiple satellite remote sensing data, and explore how the classification accuracy varies with spatial resolution, and to investigate whether the resampled remote sensing data have any influence on the scale analysis comparing with real remote image data, and which scale can most accurately represent the ground truth distribution characteristics of land cover. The results can provide a reference for selecting the optimal scale for land cover classification and a basis for scale conversion.

## 2. Study Area and Data Pre-Processing

### 2.1. Overview of the Study Area

**Figure 1:**
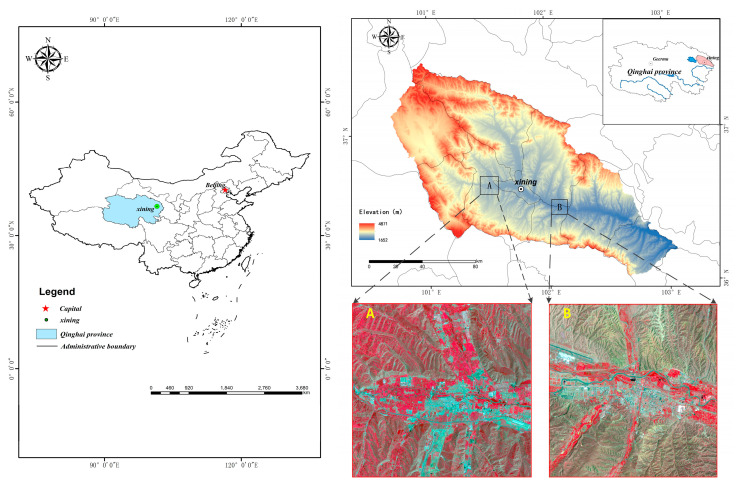
Location map of the study area.

The Huangshui River is an important first-class tributary of the upper reaches of the Yellow River, and the Huangshui basin is located in the northeast of Qinghai province, between 36°02′–37°28′ N, 100°42′–103°04′ E (Figure 1). The basin area covers 16,120 km^2^. The main cities in the basin include Xining City and Haidong City, the main population-gathering areas in Qinghai province. The land cover is greatly affected by human activities, with diverse feature types and fragmented feature patches. Xining is the capital city, as well as the political, economic, transportation, and cultural center of Qinghai province. Its administrative area includes four districts and three counties (Huangyuan County, Datong County, and Huanzhong County). Haidong City includes two municipal districts (Ledu District and Pingan District), as well as Minhe Hui and Tu Mutual Autonomous County. The topography of the whole watershed is undulating and diverse, dominated by hills and medium-high mountains. Two typical areas in two important cities in the Huangshui basin were selected for our study. One area is located in Duoba New Area of Xining, which is a key development and construction area of Xining with a complex topography and typical land cover type; and another area is located in the Ping’an District of Haidong, which is an important transportation hub of the Qinghai-Tibet Plateau and the main foreign port of Qinghai Province. Xining Caojiabao International Airport is located in this area. The topography is relatively flat, and the land cover type is typical.

### 2.2. Data Sources

The satellite images used in this study are from Chinese GF-2, French SPOT-6, ESA Sentinel-2, and U.S. Landsat-8. Among them, GF-2 is the first batch of satellites launched by the major project of China’s high-resolution earth observation system. It is the civil remote sensing satellite with the highest spatial resolution and the largest observation width developed by China. It is equipped with two high-resolution 1 m panchromatic and 4 m multi-spectral cameras. SPOT-6 was successfully launched by the French Space Center on 22 September 2012. It has an orbital altitude of 695 km and a spatial resolution of 6 m. It records images in multi-spectral blue, green, red, and near-infrared bands and 1.5 m panchromatic bands with a standard image coverage of 60 km × 60 km. Sentinel-2A is the second satellite of the European Space Agency of the European Union’s Copernicus Earth Observation Program. It was launched on 23 June 2015 for the Global Monitoring for Environment and Security program. Sentinel-2A carries a multi-spectral imager with 13 spectral bands, a strip width of 290 km, and a revisit period of 10 days. Landsat-8 is a U.S. Landsat program that was successfully launched on 11 February 2013. Landsat-8 carries the Land Imager (OLI) and the Thermal Infrared Sensor (TIRS). The OLI Land Imager includes nine bands with a spatial resolution of 30 m. The satellite sensors and their parameters are listed in Table 1 below.

## 3. Research Methodology

**Figure 2:**
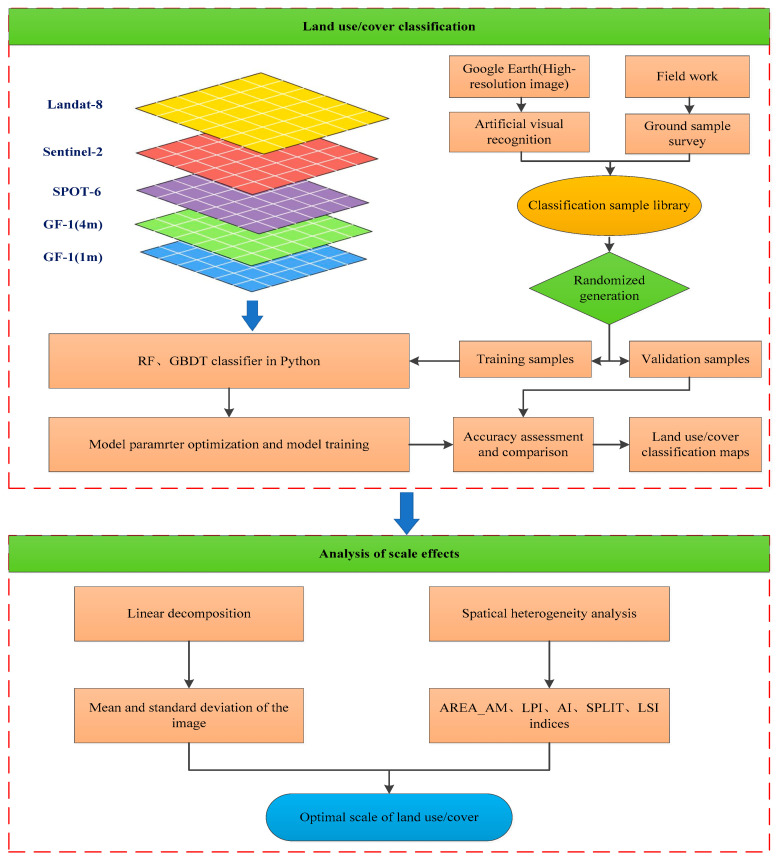
Flow chart map of land cover mapping and analysis methods.

The flow chart in Figure 2 shows the mapping and analysis methods applied in this study. Land use/cover classification and analysis of scale effects are the main steps involved. The following sections describe the analysis scheme and several relevant steps in this study in detail.

### 3.1. Ensemble Classification Methods

Ensemble learning (EL) classification methods based on multiple classifiers have been shown to be some of the most effective methods for remote sensing image classification [26,27,28]. EL trains various base classifiers separately and then combines them with related combination methods (e.g., bagging, augmentation, or voting) to produce the final classification results. Bagging ensemble methods use the same training algorithm to train several subsets, and each classifier randomly selects the training data, which means that different subsets of the same sample can be selected [29]. Then, the output of each classifier is used for voting decisions. The random forest (RF) algorithm is based on the bagging ensemble method, with a small adjustment so that the correlation between individual trees is reduced [30]. The Boosting ensemble method is an improvement on RF. The classification principle is to iteratively train a series of weak classifiers. Higher weighted attention is used to correctly classify in the next learning round, and the final result is determined by the maximum number of votes classified by the weak classifier [31]. The gradient boosting decision tree (GBDT), an algorithm among boosting ensemble methods, has been proved to be one of the most effective algorithms. It is known for its excellent performance, and recent results in many research areas have shown that it outperforms various other classifiers [32,33,34]. In this paper, we study the application of the EL method to investigate the spatially optimal scale of land cover.

#### 3.1.1. RF

RF belongs to the classification and prediction method that integrates a set of classification and regression tree (CART) decision trees. RF is the most representative bagging ensemble learning algorithm that combines bagging ensemble learning and random subspace methods to reduce overfitting [35]. In the classification process, data sets with different subsamples are randomly selected. Several decision trees are trained using different feature subsamples, and the results of the subsample decision trees are voted on to output the final classification results. RF input data does not require magnitude processing and can automatically handle missing values. It is one of the most commonly used machine learning algorithms.

#### 3.1.2. GBDT

GBDT is a Boosting ensemble machine learning method that combines multiple decision trees. GBDT is a residual model in the direction of gradient descent. It is based on the process of upgrading weak classifiers to strong classifiers. Each iteration reduces the residuals of the last iteration and constantly adjusts the weights of misclassified samples to improve the accuracy of classification. GBDT can fit the true distribution of data and has a strong generalization ability. GBDT has good overall performance due to the complementary strengths of the weak classifiers [36,37].

### 3.2. Linear Decomposition Method

The lower the spatial resolution of an image, the higher the probability that an image element contains two or more features. In ensemble learning classification, the mixed image elements are assigned to the category with the highest probability. If each hybrid image element can be decomposed and the percentage of overlay-type components to the image element can be solved, the uncertainty of classification results can be quantified, resulting in multiple hybrid image element decomposition models. The same idea can be used to calculate the percentage of various features in an image element for the classified results of low-resolution images, and the classification uncertainty of low-resolution images can be evaluated.

The principle of the hybrid image decomposition model is to decompose each hybrid image element and solve for the percentage of the overlay-type components in the decomposed image elements. The hybrid image decomposition model allows the uncertainty of classification results to be quantified. For the classified results, the same method can be used to evaluate the classification uncertainty of low-resolution images by calculating the percentage of each feature type in each image element of the low-resolution image classification results.

In this study, the area corresponding to the image element size of 1 m × 1 m after the fusion of 1 m panchromatic and 4 m multi-spectral images of GF-2 image is used as a sliding window. The land cover types contained in each window and the percentage of each type are counted on the classification results of 4 m of GF-2, 6 m of SPOT-6, 10 m of Sentinel-2, and 30 m of Landsat-8, respectively. The classification results of GF-2–4 m, SPOT-6, Sentinel-2, and Landsat-8 are only one category of cultivated land, forest land, grassland, water, built-up land, and bare land, while the GF-2 classification results of 1 m after fusion are a linear combination of each category expressed as:

(1) $$a_{1}\times c_{cultivateland}+a_{2}\times c_{forestland}+a_{3}\times c_{glassland}+a_{4}\times c_{waterland}+a_{5}\times c_{built-upland}+a_{6}\times c_{bareland}=1\;\mathrm{where}\;a_{1}+a_{2}+a_{3}+a_{4}+a_{5}+a_{6}=1\;\mathrm{and}\;a_{1},\;a_{2},\;a_{3},\;a_{4},\;a_{5},\;a_{6}\in [0,1]$$

where $a_{i}$ is the classification results of different scale images (4 m of GF-2, 6 m of SPOT-6, 10 m of Sentinel-2, and 30 m of Landsat-8) correspond to the percentage of each land cover type in GF-2 classification results of 1 m, $c_{cultivateland}$, $c_{forestland}$, $c_{glassland}$, $c_{waterland}$, $c_{built-upland}$, $c_{bareland}$ represent the land cover types: cultivated land, forest land, grassland, water, built-up land, and bareland, respectively. The value of c is 1 or 0. If there is one of the six land cover types, c is 1; if there is no one of the six land cover types, c is 0.

### 3.3. Spatial Heterogeneity Method

Spatial heterogeneity refers to the heterogeneity and complexity of the spatial distribution of ecological processes and patterns [38]. Spatial heterogeneity can generally be understood as the sum of spatial patchiness and gradient. Spatial pattern, heterogeneity, and patchiness are characteristics dependent on scale. We can define landscape indices to quantitatively describe landscape characteristics, landscape pattern information, and spatial heterogeneity, reflecting the structural composition characteristics and spatial configuration relationships of the landscape [39]. To analyze the spatial heterogeneity of different study areas, five landscape indices, namely area-weighted mean patch area (*AREA_AM*), largest patch index (*LPI*), aggregation index (*AI*), splitting index (*SPLIT*), landscape shape index (*LSI*), were selected at the landscape level in terms of area, shape, aggregation, and distribution.

*AREA_AM* is the area-weighted average patch area, calculated by the following formula:

(2) $$AREA\_AM=\frac{\sum_{i=1}^{n}w_{ij}\times a_{ij}}{\sum_{i=1}^{n}w_{ij}}$$

where $a_{ij}$ represents the area of a patch of a landscape element, $w_{ij}$ is the weight size.

*LPI* is the proportion of the largest patch in a patch type occupying the entire landscape area, in the range 0 < *LPI* ≤ 100, calculated by the following formula:

(3) $$LPI=\frac{\max a_{ij}}{A}\times 100\%$$

where $a_{ij}$ represents the area of a patch of a landscape element, and A is the total area of all landscapes.

*AI* is the aggregation index, which reflects the spatial configuration characteristics of landscape elements. The smaller *AI*, the higher the dispersion of the landscape. Conversely, the lower the dispersion of the landscape, the calculation formula is as follows:

(4) $$AI=\frac{g_{ii}}{\max g_{ii}}\times 100\%$$

where $g_{ii}$ is the number of adjacencies between different plaque types.

*SPLIT* is the landscape separation degree, which refers to the separation degree of individual distribution of different numbers of patches in a certain landscape type, calculated as follows:

(5) $$SPLIT=\frac{D_{ij}}{A_{ij}}\times 100\%$$

where $D_{ij}$ is the distance index of landscape type, $A_{ij}$ is the area index of landscape type.

*LSI* is the landscape shape index, reflecting the complexity of the overall landscape shape. The closer *LSI* is to 1, the simpler the overall landscape shape. The larger *LSI* is, the more complex. The calculation formula is as follows:

(6) $$LSI=\frac{0.25E}{\sqrt{A}}$$

where E is the total length of all patch boundaries in the landscape, A is the total area of the landscape.

## 4. Results and Analysis

### 4.1. Classification Results and Accuracy Analysis

In our study, the classification input features are 1 m and 4 m for GF-2, 6 m for SPOT-6, 10 m for Sentinel-2, and 30 m for Landsat-8 in the red, green, blue, and near-infrared bands, respectively. The classification samples are actual land cover type samples acquired by handheld GPS in the field, of which 80% are training samples, and 20% are validation samples. The classification parameters are set the same for different resolutions in the same classification method. In order to reduce the influence of other factors, the scale effects of the classification results are explored with the same inputs, the same samples, and the same parameter settings.

**Figure 3:**
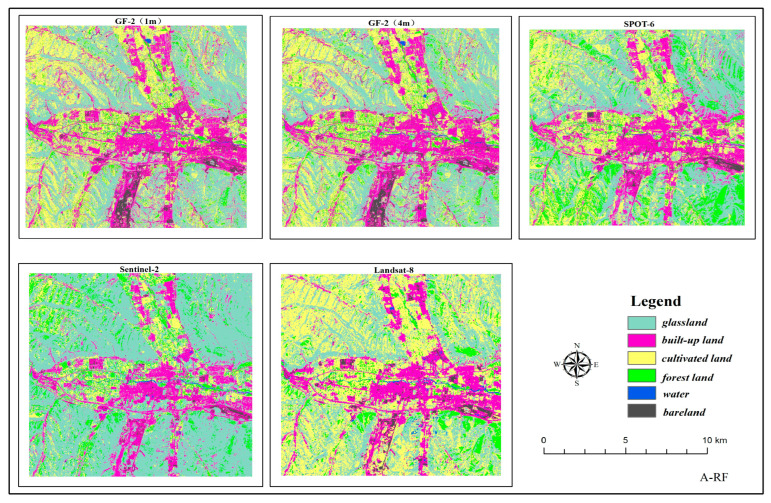

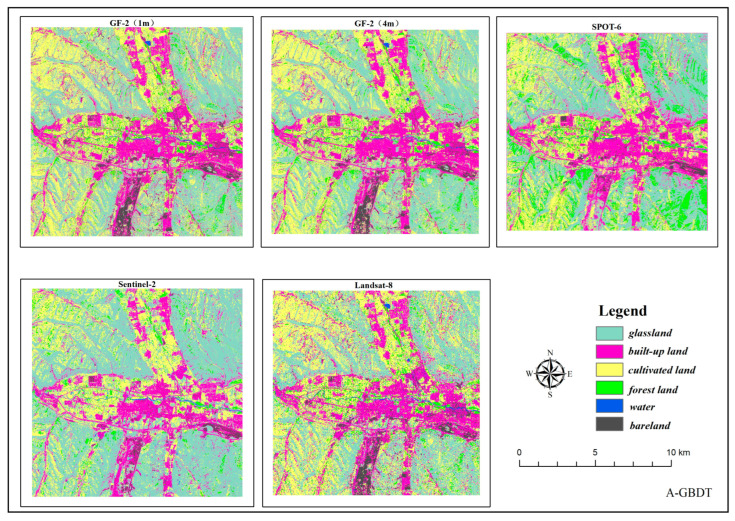
RF and GBDT land cover classification maps for Region A.

**Figure 4:**
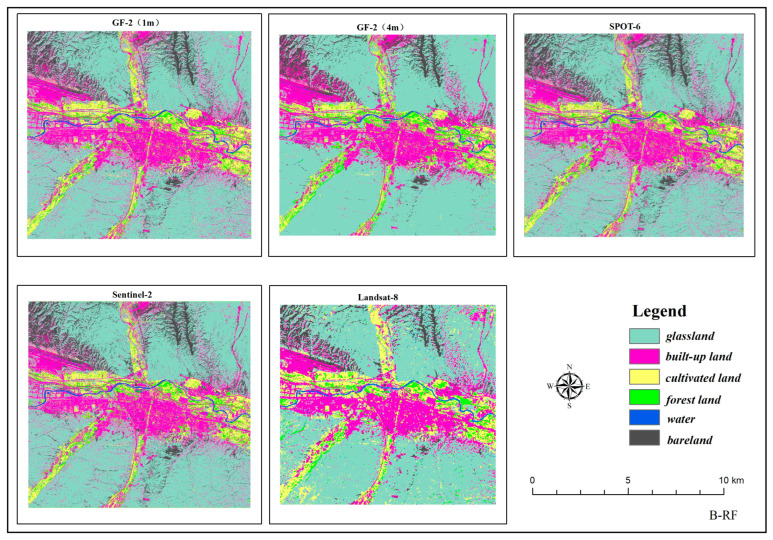

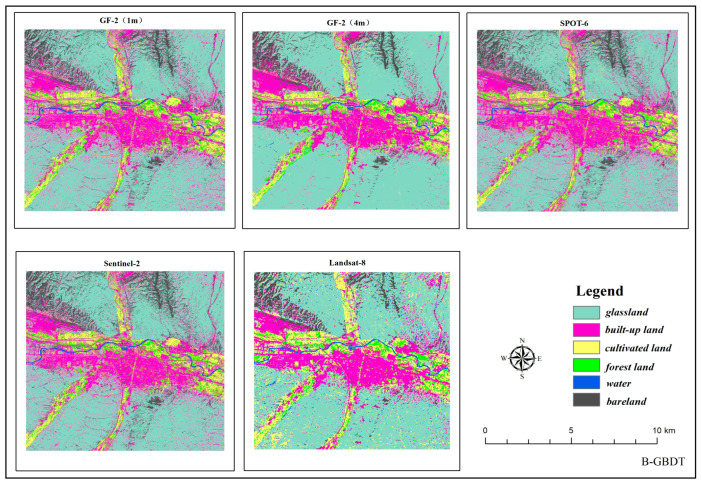
RF and GBDT land cover classification maps for Region B.

**Figure 5:**
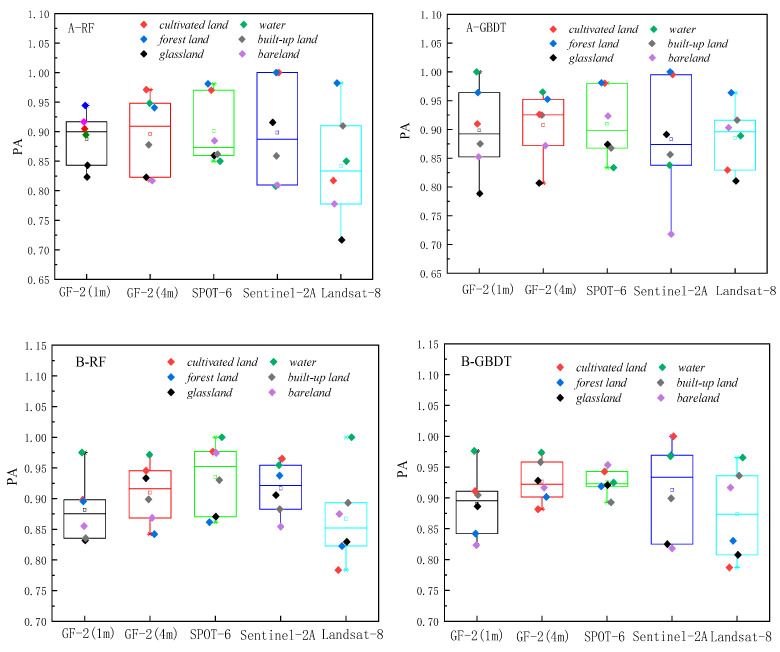
Producer’s accuracy Chart.

**Figure 6:**
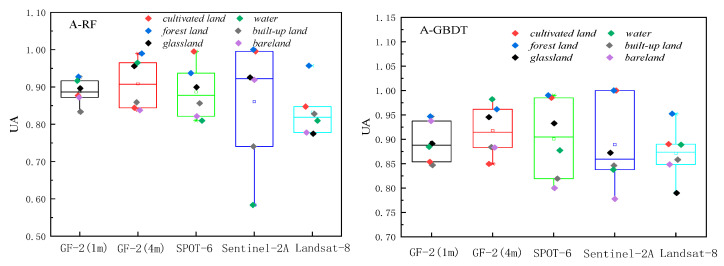

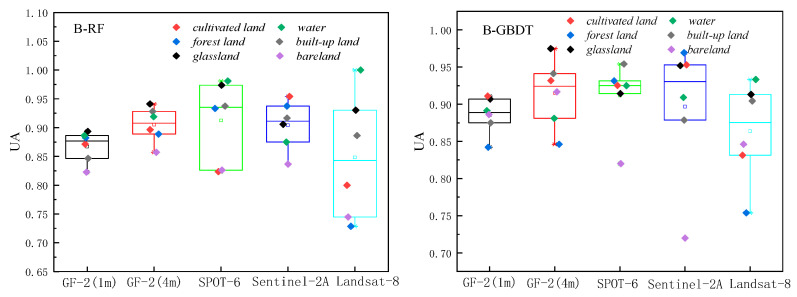
User’s accuracy Chart.

**Figure 7:**
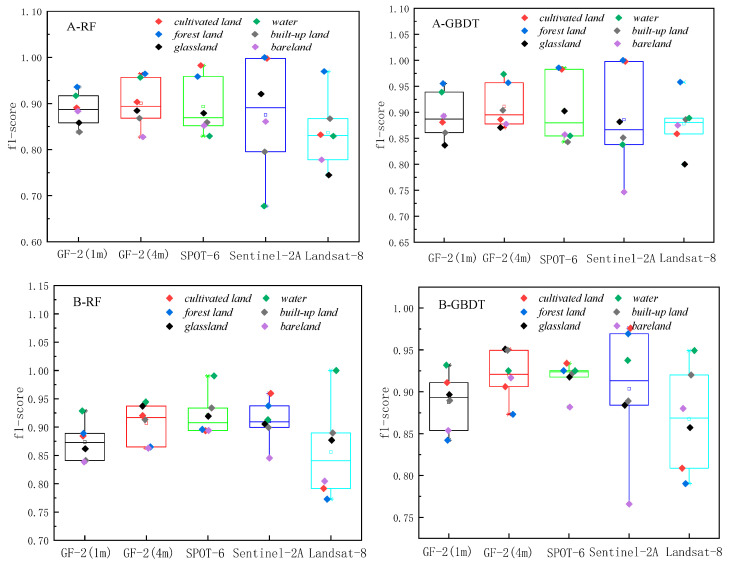
f1-score accuracy Chart.

The classification results based on RF and GBDT, two ensemble methods to classify land cover in two respective study areas, are shown in Figure 3 and Figure 4. The classification results were evaluated using the producer’s accuracy, user’s accuracy, f1-score, overall accuracy, and Kappa coefficient, as shown in Figure 5, Figure 6 and Figure 7 and Table 2.

The comprehensive analysis of the classification results of the five different spatial resolution images shows that higher spatial resolution of the images allows for better extraction features with smaller areas or sizes. For instance, small features such as reservoir pits are difficult to be extracted on images of 10 m and 30 m resolution but can be extracted on high-resolution images such as 1m and 4 m images of GF-2. Cultivated land is extracted more completely and clearly outlined on mesoscale 6 m and 10 m images, while the area of cultivated land extracted on 30 m upper images is large. The classification results are better for the small-scale resolution of feature types with larger areas and single features; for instance, grassland is better classified on 10 m and 30 m images with less pepper and better consistency.

It can be seen from Table 2 that the spatial resolution of the images largely affects the classification results of the images. For the five resolution images included in this study, the overall accuracy exceeds 84.54%. As a whole, the classification accuracy is consistent with related studies [40,41]. The effect of different classification methods on the same spatial resolution is not very significant. The classification accuracy varies by about 6% with a change of spatial resolution from 1–30 m, while the accuracy of different classification methods of the same resolution varies by about 1%, indicating that land cover classification accuracy is closely related to the spatial resolution of remote sensing images. The classification accuracy of GBDT is higher than that of RF, which indicates that the effect of boosting ensemble classification is better than that of bagging ensemble classification. The classification accuracy of the two ensemble classification methods was the highest at the spatial resolution of 4 m and 6 m and decreased when the image resolution exceeded or was smaller than 4 m. In terms of classification accuracy analyses, the optimal spatial scale of land cover for both study areas is 4–6 m, which is consistent with related studies [11,42].

The overall producer’s accuracy of GBDT is higher than that of RF. Among them, the average producer’s accuracy of GF-2 (4 m) in Region A was the highest; the average producer’s accuracy of SPOT-6 and Sentinel-2 was higher in Region B (Figure 5). The producer’s accuracy of water area is generally higher, and the producer’s accuracy of bare land is generally lower. The extreme differences between the maximum and minimum values of the producer’s accuracies of different land cover types on Sentinel-2 are larger, specifically: the extreme differences are larger in Region A than in Region B. This indicates that the distribution of the producer’s accuracies of different land cover types in Region B is more concentrated than that in Region A, and the uncertainties of different feature types in Region A are higher.

The user’s accuracy of GBDT is generally higher than that of RF. Among them, the average user’s accuracy of GF-2 (4 m) in Region A is the highest; the average user’s accuracy of SPOT-6 and Sentinel-2 is higher in Region B (Figure 6). The user’s accuracy of forest land in Region A is generally higher; the user’s accuracy of unutilized land in Region B is lower. The larger extreme difference between Region A and Region B indicates that the distribution of user accuracy for different land cover types in Region B is more concentrated than that in Region A. The uncertainty of different feature types in Region A is higher.

The f1-score is the average of producer accuracy and user accuracy. The average f1-score accuracy of GF-2 (4 m) in Region A is the highest; the average f1-score accuracy of SPOT-6 and Sentinel-2 is higher in Region B; the f1-score of Landsat-8 is lower in both areas (Figure 7). Based on the classification accuracy, it can be concluded that the spatial resolution dependence of the watershed on the image is low, while the dependence on woodland and cropland is high. The accuracy of GF-2 (4 m) is better in Region A, and the accuracy of SPOT-6 and Sentinel-2 is better in Region B. Therefore, the uncertainty of Region A is higher than that of Region B, and the optimal scale accuracy of Region B corresponds to a lower spatial resolution than the optimal scale accuracy of Region A.

### 4.2. Optimal Scale Analysis Based on Linear Decomposition

**Figure 8:**
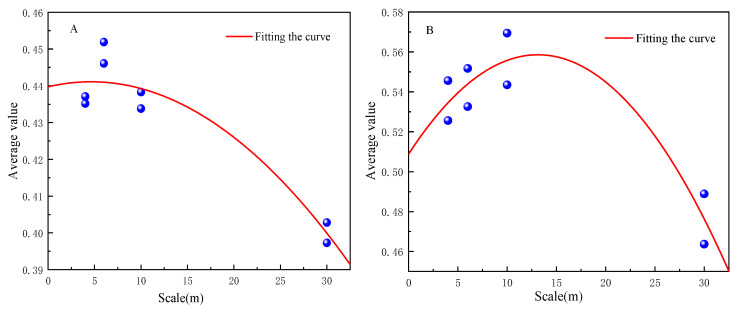
Upper scale fit of Region A and Region B.

Based on the linear decomposition method, the 1 m classification results of GF-2 fusion were used as a reference for Landsat-8, Sentinel-2, and SPOT-6 classification results. The fitted equations and related parameters were established using the curve fitting method and are summarized in Table 3. The coefficients of determination are greater than 0.85 (*p* < 0.01) in both study areas. The fitted curves are shown in Figure 8.

The scale effect of the fitted curves based on the linear decomposition method in Region A and Region B shows that the mean value of the decomposition tends to increase and then decrease as the scale increases (Figure 8). The optimal classification scale of Region A is 5 m, and the optimal scale of Region B is 13 m. The optimal scale of decomposition is different for different study areas, mainly because of the different distribution patterns of topographic and geomorphological complexity and land cover types in the two areas, and therefore the spatial heterogeneity is different. Region A has a more complex topography and more fragmented patches. Region B is located in Xining Caojiabao Airport, with relatively flat topography and regular feature patches, boundaries, and shapes. Therefore, the optimal land cover scale of Region B is larger than that of Region A. That is, the spatial resolution of remote sensing images used for the classification of Region B is larger than that of Region A.

**Figure 9:**
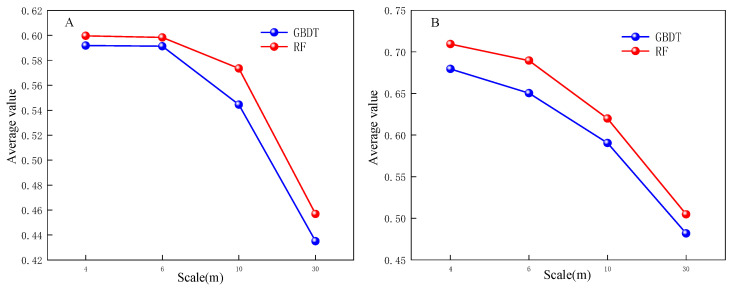
Linear decomposition after resampling on Region A and Region B.

To compare the scale effect of image resampling on land cover, resampling was performed based on the same scene image. To compare data sources and maintain maximum spectral information, the sampling method chosen used the nearest neighbor sampling method with the least information loss. This has less impact on the amount of image information and is especially suitable for resampling before classification [43]. Therefore, the GF-1 fused 1 m image was resampled into 4, 6, 10, and 30 m by the nearest neighbor sampling method. The results of the linear decomposition based on resampling are shown in Figure 9.

It can be seen that the linear decomposition result based on the classification after resampling is not sensitive to the scale (Figure 9). The linear decomposition gradually decreases as the scale rises. The linear decomposition value and scale of the classification after resampling do not show a trend of first increasing and then decreasing, probably because resampling introduces errors.

### 4.3. Spatial Heterogeneity Analysis

Land cover classification of remote sensing images is mainly influenced by two factors: mixed image elements and spatial heterogeneity. Mixed image element decomposition shows that the choice of the optimal scale for land cover classification is related to the study area. The more complex the study area, the higher the resolution or the larger the scale of the image for classification. To analyze the spatial heterogeneity of different study areas, five landscape indices, namely AREA_AM, LPI, AI, SPLIT, and LSI, were selected from the aspects of the area, shape, aggregation, and distribution at the landscape level. The landscape indices values were calculated based on the landscape pattern analysis software Fragstats4.2. The calculation results in two areas based on two classification methods are shown in Table 4.

From the above tables, the average patch area of Region B is larger than that of Region A. The maximum patch area of Region B is twice as large as that of Region A. The proportion of the largest patches to the landscape area in Region B is larger than that in Region A, the aggregation is better, and the shape is more regular. Region A is more spatially heterogeneous than Region B. Therefore, the classification of land cover types in Region B corresponds to a larger scale, consistent with the results of linear decomposition.

According to our results, the optimal classification result is not necessarily the optimal land cover scale. The classification results are the expression of macroscopic feature patterns, and factors such as shape, patchiness, and aggregation separability associated with feature characteristics determine the optimal scale of land cover. The more fragmented and complex the features are, the smaller the optimal land cover scale needs to be to finely represent the feature distribution.

## 5. Discussion

### 5.1. Discussion of Classification Methods

RF and GBDT are the most popular bagging and boosting ensemble methods based on decision trees. In our study, the GBDT model outperforms the RF model due to the different ensemble of trees in the ensemble methods. RF uses bagging (bootstrap resampling) method to construct different training sets and determines the final classification result using the maximum number of votes for the results of different datasets. GBDT uses gradient boosting to create a tree based on the residuals of the previously created tree. Both RF and GBDT have advantages, such as dealing with nonlinearities and limiting overfitting. Dietterich [44] compared bagging and boosting ensembles and found that the noise in the boosting dataset is less than that in the bagging dataset, which means that boosting is sensitive to the noise in the data. The signal-to-noise ratios of data at different scales are different, but in this study, RF and GBDT are better in GF-2, SPOT-6, Sentinel-2, and Landsat-8 classifications. The ensemble classifier has complementary advantages over a single classifier and can improve classification accuracy, in accordance with Feng et al. [45].

Yang et al. [46] used three methods, ISODATA, MLC, and SVM, to classify land cover in the Poyang Lake region, a region with strong spatial heterogeneity during the dry period. Their results indicate that different land cover classification methods may lead to different classification results for the same remote sensing data. In the same geographical area, the classification results may be different when using the same classification methods for land cover classification of remote sensing data with different resolutions. Even if the same classification method is used to classify land cover data with different resolutions in the same geographical area, the classification results may be different. By contrast, in our study, the classification results from RF and GBDT show more consistent results on different remote sensing data, and both SPOT-6 and GF-2 (4 m) have higher classification accuracy in two different areas. Therefore, the advantages of the ensemble method are better for multi-scale data usability.

### 5.2. Uncertainty Analysis of Classification

Determining the optimal classification system is the premise and foundation of land cover classification. A suitable classification system should consider both the actual situation of the study area and the spectral and spatial resolution of image data. At present, the national land use/cover classification system for remote sensing monitoring gives six major categories: cultivated land, forest land, grassland, water area, urban and rural industrial and mining residential construction land, and bare land. Our study areas are located in a high altitude, hilly, middle, and high mountainous terrain area and consider only six major categories of land cover classification. A more detailed classification may cause more complicated linear decomposition and spatial heterogeneity. Therefore, the conclusions drawn in this paper are applicable to the six major categories of land cover classification, and more detailed land classification may require further study.

**Figure 10:**
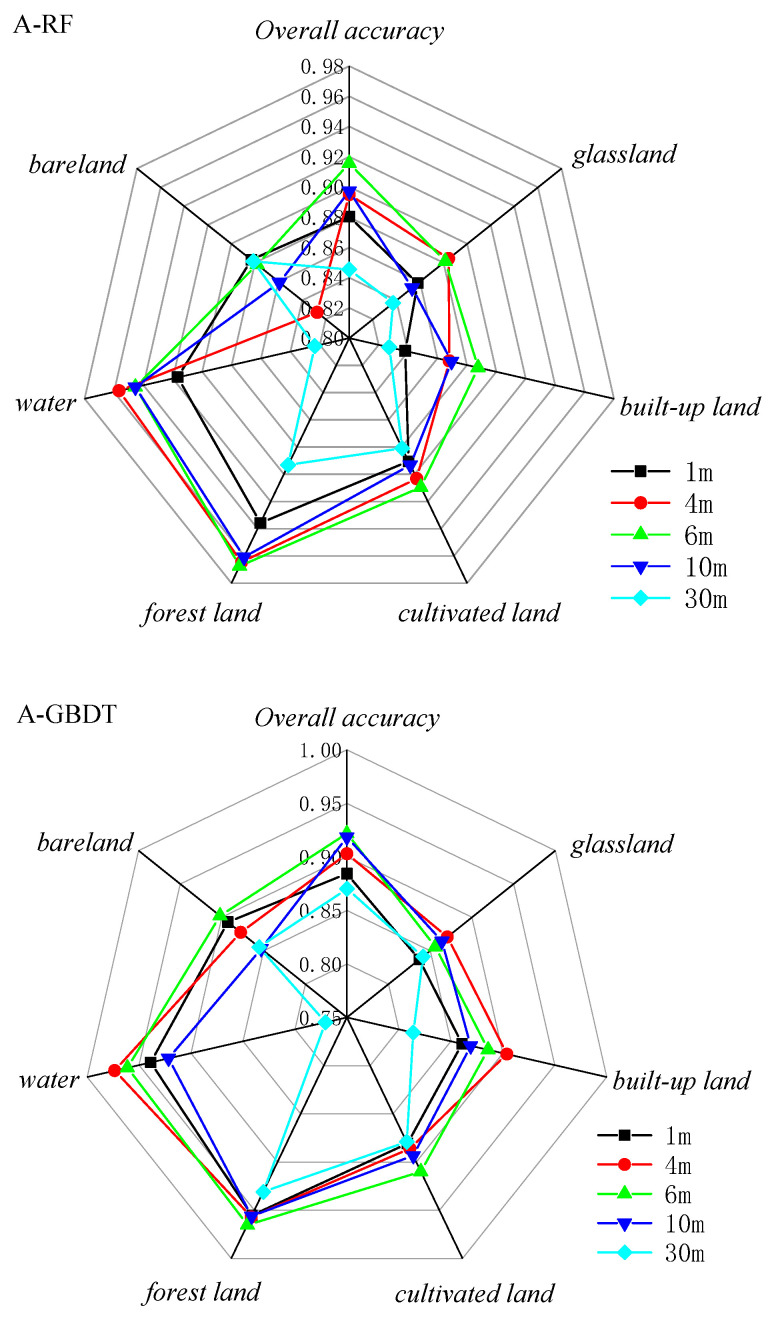

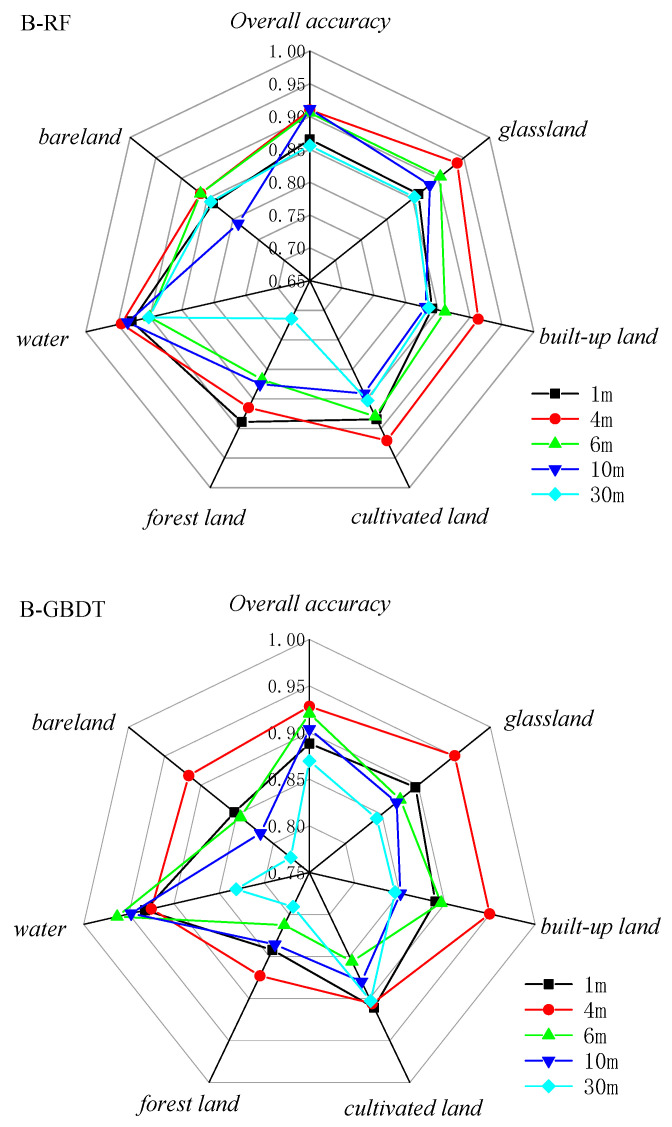
Scale fit after resampling in regions A and B.

From the analysis of classification accuracy after resampling, the overall classification accuracy of real images with different resolutions and the f1-score of each category after resampling in study Region A and Region B are shown in Figure 10. From the figure, it can be seen that the overall classification accuracy and consistency between each category are not very good. The consistency in study Region B is better than that in study Region A. The greater the regional heterogeneity, the greater the effect on the images after resampling. Combining the analysis of Region A and Region B, the consistency was higher on urban and rural industrial and mining residential construction land and forest land, and the uncertainty was larger in other categories, which is basically similar to the findings of Xu et al. [47]. It further indicates that resampling increases the uncertainty of the classification.

### 5.3. Uncertainty Analysis Based on Linear Model

**Figure 11:**
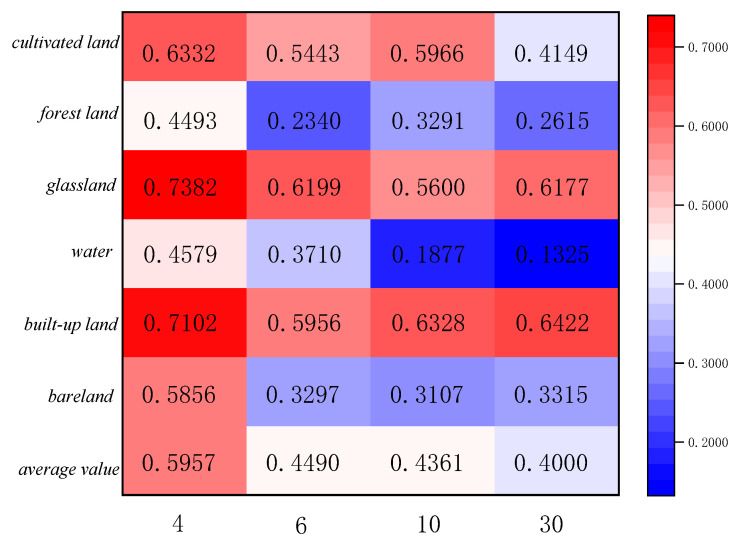
Class mean statistics of classification results at different scales for Region A.

**Figure 12:**
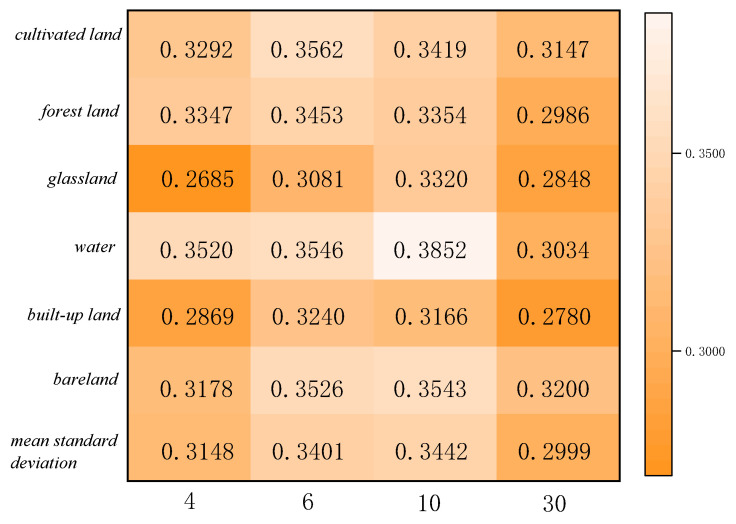
Category standard deviation statistics of classification results at different scales for Region A.

**Figure 13:**
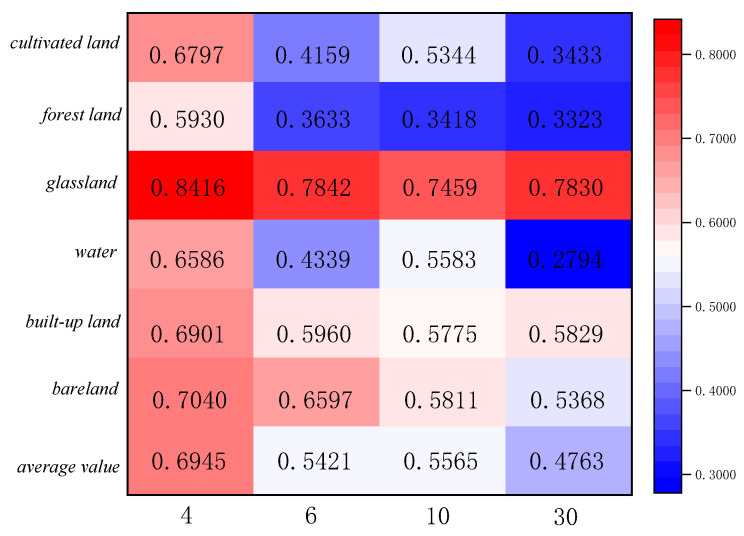
Class mean statistics of classification results at different scales for Region B.

**Figure 14:**
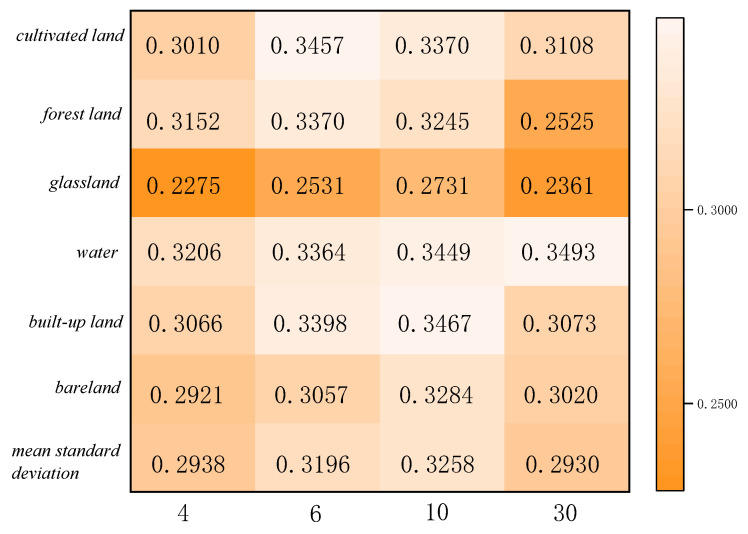
Category standard deviation statistics of classification results at different scales for Region B.

The statistics of feature categories in Region A and Region B for the two ensemble learning classification methods are shown in Figure 11, Figure 12, Figure 13 and Figure 14. The table refers to the mean and standard deviation of the image elements of each category on the SPOT-6, Sentinel-2, and Landsat-8 classification images. This is similar to the misclassification cost (MCC) introduced by Defries et al. [48] for land cover classification accuracy evaluation, where the degree of misclassification is different between categories.

According to the statistical results of regions A and B based on the linear decomposition method, it can be seen that with the decrease in spatial resolution, the mean results of different ground object types decrease, indicating that the decrease in spatial resolution increases the number of mixed pixels, the spatial heterogeneity, and the spatial uncertainty.

In the two study areas, the proportions of cropland, grassland, urban and rural industrial and mining residential construction land, and bare land are larger, and their consistency of classification results on SPOT-6, Sentinel-2, and Landsat-8 images is higher. There is less uncertainty in the attribution of image categories for these four features. The mean value of water is lower because the areas under water are smaller and distributed in strips, corresponding to a smaller scale. The mean value of GF-2 (4 m) is the largest in both study areas, 0.5957 and 0.6945, indicating 40% uncertainty in Region A and 30% uncertainty in Region B. The mean standard deviation is around 0.3, indicating fewer fluctuations and more reliable mean decomposition.

## 6. Conclusions

In this paper, we explored the scale effect of land cover classification from mixed image element decomposition and spatial heterogeneity using multi-source and multi-scale satellite remote sensing data and came to the following conclusions:

(1) GF-2, SPOT-6, Sentinel-2, and Landsat-8 images with different spatial resolutions based on GBDT and RF were used for classification studies, and GF-2 and SPOT-6 had the best classification results. Therefore, the optimal scale based on classification accuracy is 4–6 m.
(2) The optimal scale based on linear decomposition is related to the study area, and the optimal scale is different for different study areas.
(3) The optimal scale of land cover classification is related to spatial heterogeneity. The more fragmented and complex the space, the smaller the scale needed.
(4) Images based on resampling do not reflect the characteristics of the actual scale images well, are insensitive to scale effects and increase the uncertainty of the classification.
(5) The best classification result is not necessarily the optimal land cover scale. The classification result is only a representation of the macroscopic feature pattern. Factors such as shape, patchiness, and aggregation separability associated with feature characteristics determine the optimal land cover scale.

## Figures and Tables

**Table 1:** Satellite sensors and their parameters.

Satellite Sensor	Country	Spatical Resolution (m)	Wave Band	Wavelength Coverage (μm)	Acquisition Data
GF-2	China	1, 4	Blue, Green, Red, Near-infrared (R, G, B, NIR)	0.45~0.52/0.52~0.59 0.63~0.69/0.77~0.89	21 July 2017
SPOT-6	France	6		0.455~0.525/0.530~0.590 0.625~0.695/0.760~0.890	19 July 2017
Sentinel-2A	ESA	10		0.440~0.538/0.537~0.582 0.646~0.684/0.760~0.890	27 July 2017
Landsat-8	America	30		0.450~0.515/0.525~0.600 0.630~0.680/0.845~0.885	11 September 2017

**Table 2:** Classification of overall accuracy and Kappa coefficient of the two classification methods.

Region	Satellite Sensor	RF		GBDT	
		OA (%)	Kappa	OA (%)	Kappa
A	GF-2 (1 m)	88.03	0.85	88.43	0.86
	GF-2 (4 m)	89.50	0.87	90.29	0.88
	SPOT-6	91.56	0.89	92.19	0.90
	Sentinel-2A	89.73	0.87	91.85	0.89
	Landsat-8	84.54	0.80	87.01	0.84
B	GF-2 (1 m)	86.53	0.83	88.83	0.86
	GF-2 (4 m)	91.01	0.89	92.81	0.91
	SPOT-6	90.57	0.90	92.05	0.90
	Sentinel-2A	91.17	0.89	90.39	0.88
	Landsat-8	85.57	0.82	86.96	0.83

**Table 3:** Fitting model and related parameters.

Region	Fitting Equation	*R* ^2^	*p*
A	$y=0.4397+0.5906\times 10^{-3}\times x-0.6393\times 10^{-4}\times x^{2}$	0.911	0.002
B	$y=0.5088+7.5814\times 10^{-3}\times x-0.2888\times 10^{-3}\times x^{2}$	0.877	0.005

**Table 4:** Landscape index values based on RF and GBDT in Region A and Region B.

Region	Satellite Sensor	RF					GBDT				
		AREA_AM	LPI	AI	SPLIT	LSI	AREA_AM	LPI	AI	SPLIT	LSI
A	GF-2 (1 m)	361.93	12.39	88.03	23.47	552.93	331.02	11.64	81.82	25.66	839.41
	GF-2 (4 m)	605.55	12.30	81.13	30.32	321.02	599.21	12.06	77.11	30.63	388.99
	SPOT-6	693.96	10.93	84.47	26.50	176.83	761.20	12.36	79.38	24.15	234.33
	Sentinel-2A	1488.94	16.36	83.58	12.33	112.55	1012.56	12.61	76.69	18.13	159.15
	Landsat-8	951.74	17.39	64.86	19.28	80.50	694.40	9.27	62.58	26.42	85.63
B	GF-2 (1 m)	533.92	15.88	89.60	13.86	534.97	531.19	15.92	83.43	13.93	713.88
	GF-2 (4 m)	1352.46	22.29	89.78	9.41	145.59	1412.41	23.34	86.78	9.01	187.78
	SPOT-6	1421.05	24.66	87.88	8.96	115.28	1626.08	23.48	83.92	9.34	152.53
	Sentinel-2A	1660.92	27.40	85.12	7.67	85.21	1586.00	26.46	80.11	8.03	113.43
	Landsat-8	1401.22	23.92	75.64	9.09	47.02	1314.06	23.48	72.47	9.50	52.94

## Data Availability

The data presented in this study are available upon request from the first author.
